# Total arch replacement using frozen elephant trunk technique with Frozenix for distal aortic arch aneurysms

**DOI:** 10.1093/icvts/ivac038

**Published:** 2022-02-18

**Authors:** Chiho Tokunaga, Yu Kumagai, Fumiya Chubachi, Yuto Hori, Akitoshi Takazawa, Jun Hayashi, Toshihisa Asakura, Ryota Ishii, Hiroyuki Nakajima, Akihiro Yoshitake

**Affiliations:** 1 Department of Cardiovascular Surgery, Saitama Medical University, International Medical Center, Saitama, Japan; 2 Department of Biostatistics, Faculty of Medicine, University of Tsukuba, Ibaraki, Japan

**Keywords:** Total arch replacement, Frozen elephant trunk technique, Distal aortic arch aneurysm

## Abstract

**OBJECTIVES:**

Total arch replacement (TAR) using an endovascular approach has been initially introduced as the frozen elephant trunk technique (FET). In our institute, TAR using the FET with Frozenix has been used as the first-line treatment for distal aortic arch aneurysms since 2014. This study aimed to evaluate the early and long-term outcomes and demonstrate the efficacy of this procedure.

**METHODS:**

Between 2014 and 2021, 121 consecutive patients were treated with TAR using the FET with Frozenix for distal aortic arch aneurysms. Early and long-term outcomes were retrospectively analysed.

**RESULTS:**

The 30-day mortality rate was 2.5% (3/121). Of postoperative complications, paraplegia due to spinal cord injury occurred in 2 (1.7%) patients, stroke in 12 (9.9%) and acute renal failure in 10 (8.3%). At follow-up, 23 secondary aortic interventions were required and 8 (6.6%) patients underwent intended secondary thoracic endovascular aortic repair for residual descending aortic aneurysm. Late and aortic-related deaths occurred in 16 (13.2%) and 4 (3.3%) patients, respectively. The overall long-term survival rates at 1, 3 and 5 years were 87.6%, 83.1% and 65.4%, respectively, while the rates of freedom from aortic-related death at 1, 3 and 5 years were 95.7%, 95.7% and 84.8%, respectively.

**CONCLUSIONS:**

TAR using the FET with Frozenix for distal aortic arch aneurysms has acceptable early mortality and morbidity. Spinal cord injury and paraplegia occur less frequently than previously reported. The technique has satisfactory long-term survival and freedom from aortic-related death.

**Figure:**
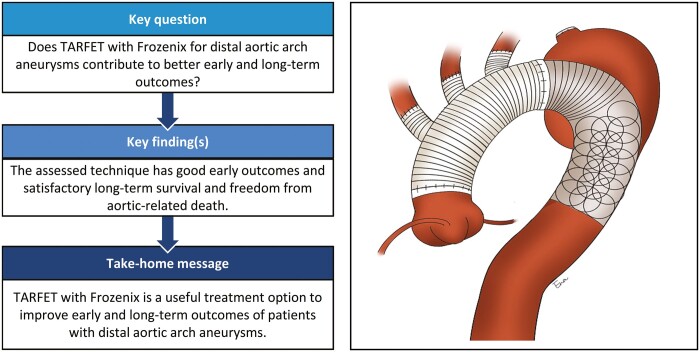

## INTRODUCTION

The number of patients with extensive aortic degenerative disease involving the distal aortic arch has increased in the recent ageing society [1, 2]. In this context, distal aortic arch aneurysm remains a great challenge for cardiovascular surgeons because of the complex surgical strategy for aortic arch repair and limited access to the aneurysm at the descending aortic arch from median sternotomy [3]. Multiple surgical strategies have been reported; the 1-stage approach is 1-stage aortic arch replacement with wide skin incision [4, 5], while the 2-stage approach is a staged aortic surgery with elephant trunk technique following second-stage descending aorta replacement [6, 7]. More recently, hybrid treatment was developed, and it combines open surgical and endovascular techniques using a stent-graft [8]. Kato *et al.* [9] first reported the combination of conventional total arch replacement (TAR) with an endovascular technique named ‘open stent grafting’ in 1996. In 2003, Karck *et al.* [10] reported TAR with stent-graft insertion into the descending aorta as the frozen elephant trunk technique (FET) for descending aortic aneurysm or chronic aortic dissection. Since then, the indications for the FET have been expanded widely, and this new approach became an alternative strategy for extended aortic diseases because it allows complete surgical treatment of combined aortic lesions during a single operation through a median sternotomy [11–14]. TAR using the FET (TARFET) for acute aortic dissection has been gaining interest and popularity, as this procedure helps provide primary entry closure at the descending thoracic aorta with feasible long-term outcomes [15, 16]. However, there are limited data on the TARFET for distal aortic arch aneurysms compared with acute or chronic aortic dissection, and long-term outcomes have not been fully elucidated.

The Japan-made commercialized FET prosthesis (J Graft Frozenix, Japan Lifeline, Tokyo) was first launched in 2014 [1, 17]. The TARFET using a tetrafurcated tube graft for TAR and Frozenix prosthesis for the FET has been induced as the first-line treatment for distal aortic arch aneurysms since 2014 in our institute.

The objective of this study was to evaluate the early and long-term outcomes of the TARFET with Frozenix prosthesis for distal aortic arch aneurysms and demonstrate the efficacy of this procedure.

## MATERIALS AND METHODS

### Ethical statement

The study protocol was approved by the Ethics Committee of the Saitama Medical University International Medical Center (2021-093). Given the retrospective nature of the research, the Ethics Committee waived the need for written patient informed consent.

### Patients and study design

Consecutive 159 patients who were treated for distal aortic arch aneurysms at our institution from 2014 to 2021 were enrolled in this study.

A distal aortic arch aneurysm was defined as an aneurysm located at the aortic arch and extending from the root of the left subclavian artery to the descending thoracic aorta. The strategy for distal aortic arch aneurysms in our institute is as follows: (i) open surgery is considered a primary treatment rather than endovascular surgery if there is no contraindication, (ii) the TARFET is the first-line treatment in open surgery if 1-stage surgery with the TARFET can achieve complete coverage of the descending aortic aneurysm above the Th8 and (iii) if the FET distal landing to cover the whole distal aortic arch aneurysm would be below the Th8, the 2-staged surgery with the conventional elephant trunk technique following either descending aorta replacement or secondary thoracic endovascular aortic repair (TEVAR) was preferred. In addition, if the morphology of the descending aorta is not suitable for placing the distal end of the FET, for example, shaggy aorta or dilated diameter over the Frozenix size, the conventional elephant trunk technique following secondary intervention for the downstream aorta was performed.

### Operative technique

All patients underwent TARFET through median sternotomy. Direct cannulation of the ascending aorta was selected mainly, and one side of the femoral artery was chosen for additional arterial cannulation. After cardiopulmonary bypass was established, the patients were cooled until their rectal or bladder temperatures reached 26°C. Under circulatory arrest, selective cerebral perfusion was established using balloon-tipped cannulas from the inside of the aortic arch to the brachiocephalic, left carotid and left subclavian arteries.

The FET prosthesis, Frozenix, consists of a Dacron polyester fabric vascular prosthesis with nitinol stents affixed on the inner aspect. The delivery system consists of a malleable rod that can be advanced into the descending aorta. As the stent has a constant length, the distal end of the stent-graft can be fixed as required. The Frozenix is available with lengths of 60, 90, 120 and 150 mm of the stented portions and diameters of 21–39 mm. Preoperative multiplanar reconstruction computed tomography was performed routinely, and the distances between the planned distal anastomosis of the tetrafurcated tube graft for TAR and the target distal end of the FET prosthesis were measured. The FET prosthesis length was selected to cover the straight descending aorta above the thoracic vertebra 8 level, and the FET prosthesis diameter was selected on 110–120% diameter of the descending aorta. The aortic arch was normally transected between the left common carotid artery and the left subclavian artery as zone 2, while zones 0, 1 and 3 anastomoses were chosen depending on the condition of the aortic arch. The arch side orifice of the supra-aortic vessels was closed by continuous suture with a felt strip. The FET prosthesis was inserted into the descending aorta, and the distal stump was made by continuous suture reinforced with an outer felt strip. To prevent embolism by debris at the time of FET deployment, distal perfusion from the femoral artery was performed, depending on the severity of the shaggy aorta. Another 4-branched graft was anastomosed to the stump under open distal anastomosis fashion, and antegrade systemic perfusion was then started from the fourth branch of the graft. After proximal anastomosis, the left subclavian, left carotid and brachiocephalic arteries were anastomosed to each graft branch in an end-to-end fashion.

Cerebrospinal fluid drainage was not routinely performed before the TARFET at our institute.

### Definitions

Early mortality was defined as 30-day mortality, and late mortality was defined as all-cause mortality from the time of the TARFET performed. Late aortic-related death was defined as a procedure- or disease-related death after 30 days of the TARFET including aneurysm rupture or aortic infection.

### Endpoints and follow-up

The primary endpoints were 30-day mortality and late mortality. The patients were followed up at the outpatient unit of our institute, and a computed tomography scan was performed to evaluate aneurysm remodelling on a yearly basis. In patients who underwent follow-up in another hospital, contact with the primary physicians or telephone interviews were conducted. Loss to follow-up was defined as patients who were lost to follow-up within 30 days after discharge [18]. The follow-up index was calculated for each patient [18].

### Statistical analysis

Continuous variables are presented as mean [standard deviation (SD)] if normally distributed and median with interquartile range for non-normal distributions. Within-subject changes of aneurysm diameter were compares using the paired *t*-test. A *P*-value < 0.05 was considered significant. Missing data were treated with pairwise deletion. Long-term outcomes were estimated using the Kaplan–Meier analysis. The probabilities of secondary intervention were estimated from cumulative incidence function. All data were analysed using SPSS version 24.0 software (IBM Corp., Armonk, NY, USA) and SAS version 9.4 (SAS Institute Inc., Cary, NC, USA).

## RESULTS

**Figure 1:**
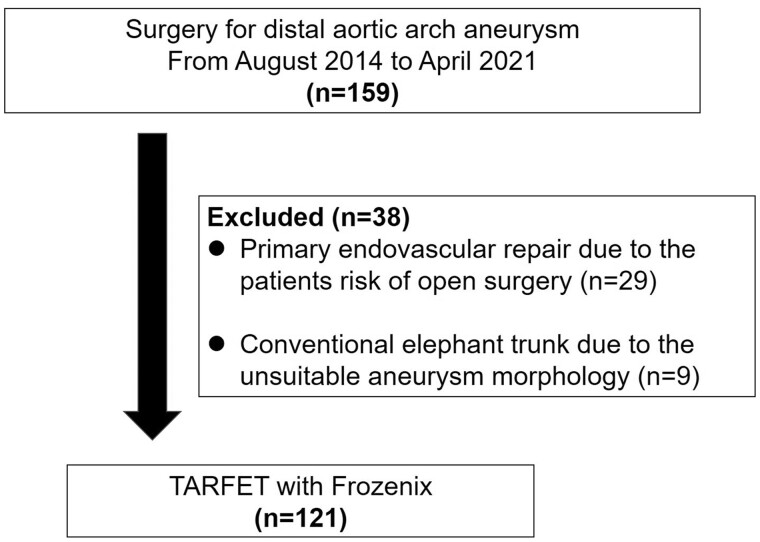
Flow chart outlining patient selection. TARFET: total arch replacement using frozen elephant trunk technique.

In total, 159 patients were treated for distal aortic arch aneurysms at our institution from 2014 to 2021. Of them, 29 patients were excluded because they underwent primary endovascular procedures according to the patient’s risk of open surgery. Nine patients underwent conventional elephant trunk procedures due to the unsuitable aneurysm morphology for the TARFET. The remaining 121 (76.1%) patients underwent the TARFET with Frozenix (Fig. 1).

Preoperative characteristics of 121 patients who underwent the TARFET with Frozenix are outlined in Table 1, and operative characteristics are summarized in Table 2. In this study, 30 (24.8%) patients had coronary artery bypass grafting simultaneously with the TARFET due to concomitant coronary artery diseases.

**Table 1:** Preoperative patient characteristics

Variable	*n* = 121
Age (years)	74.3 (SD: 6.6)
Male sex	103 (85.1)
Hypertension	100 (82.6)
Chronic obstructive pulmonary disease	20 (16.5)
Diabetes mellitus	20 (16.5)
Chronic kidney disease (Cre > 1.5 mg/dl)	73 (60.3)
Haemodialysis	7 (5.8)
Cerebral infarction	19 (15.7)
Coronary artery disease	28 (23.1)
EF (%: range)	63.5 (SD: 11.6)
Previous sternotomy	7 (5.8)

Values are presented as number (%) or mean (SD).

EF: ejection fraction.

**Table 2:** Operative characteristics

Variable	*n* = 121
Aneurysm characteristics	
Saccular/fusiform aneurysm	71 (58.7)/50 (41.3)
Ruptured aneurysm/emergency	8 (6.6)
Aneurysm diameter (mm)	57.3 (SD: 9.6)
Operative characteristics	
Cardiopulmonary bypass (min)	194.7 (SD: 39.9)
Selective cerebral perfusion (min)	120.7 (SD: 28.9)
Aortic cross-clamp (min)	104.2 (SD: 29.3)
Circulatory arrest (min)	56.3 (SD: 13.4)
Minimum rectal temperature	26.1(SD: 1.3)
Concomitant procedures	
Coronary artery bypass grafting	30 (24.8)
Aortic valve replacement	6 (5.0)

Values are presented as number (%) or mean (SD).

**Figure 2:**
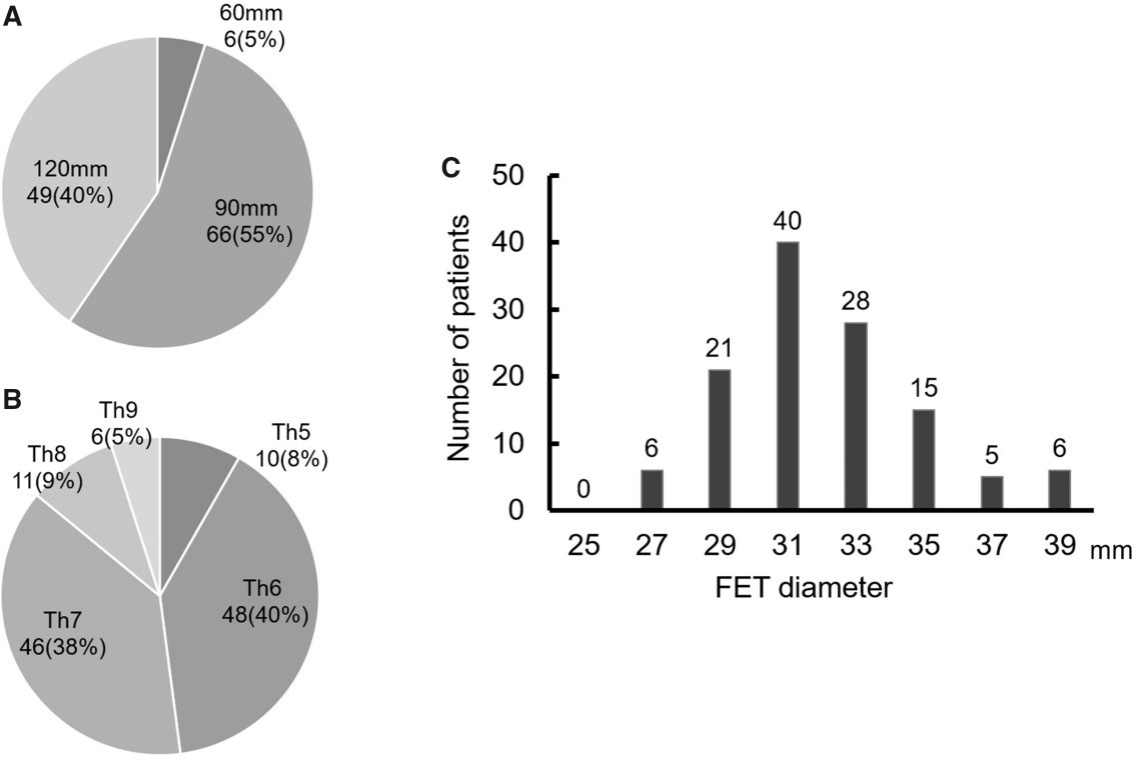
FET length (**A**), distal landing zone of the FET (**B**), and distribution of FET diameter (**C**). The FET length was selected to cover the straight descending aorta above the Th8 level (87% of FETs were placed above the Th8). FET diameter was selected based on 110–120% diameter of the distal descending aorta. FET: frozen elephant trunk.

The length and diameter of FET prosthesis Frozenix used in patients with distal aortic arch aneurysms are shown in Fig. 2. Distal anastomosis was performed at zone 0 in 12 (9.9%) patients, zone 1 in 16 (13.2%), zone 2 in 82 (67.8%) and zone 3 in 11 (9.1%). The distal arch aneurysm was excluded in 191 (98.4%) patients.

Early and late operative outcomes are presented in Table 3. The mean follow-up period was 24.1 (SD: 20.6; ranged, 1–80) months. A total of 8 (6.6%) patients were lost to follow-up, and the median follow-up index was 1.0 (1.0, 1.0) [18]. During follow-up, the excluded distal aortic aneurysm was remodelled, and the mean diameter of the aneurysm significantly decreased from 57.3 mm (SD: 9.6) to 50.4 mm (SD: 11.1) (*P* < 0.01).

**Table 3:** Early and late operative results

Variable	*n* = 121
30-day mortality	3 (2.5)
Morbidity	
Transient neurological deficit	7 (5.8)
Permanent neurological deficit	5 (4.1)
Acute renal failure	10 (8.3)
Pneumonia	4 (3.3)
Spinal cord injury/paraplegia	2 (1.7)
Late mortality	16 (11.6)
Aortic-related death	4 (3.3)
Non-aortic-related death	12 (9.9)
Late aortic event	23 (19.0)
Intended secondary aortic intervention	8 (6.6)
Non-intended secondary aortic intervention	15 (12.4)

Values are presented as number (%).

Overall, the 30-day mortality rate was 2.5% (3 patients). The causes of death were sepsis, low output syndrome and stroke (each in 1 patient). Regarding postoperative complications, paraplegia due to spinal cord ischaemia occurred in 2 (1.7%) patients, postoperative stroke in 12 (9.9%), including transient and permanent neurological deficits in 7 (5.8%) and 5 (4.1%). Acute renal failure requiring transient renal replacement therapy was observed in 10 (8.3%) patients, and further induction of permanent haemodialysis was required in 1 (0.8%) patient, while the other 9 (7.4%) patients recovered and were weaned from renal replacement therapy. Respiratory failure due to pneumonia, pneumothorax and repeated atelectasis occurred in 4 (3.3%), 2 (1.7%) and 2 (1.7%) patients, respectively. Furthermore, tracheostomy was required in 2 (1.7%) patients.

During follow-up, 23 secondary aortic interventions were performed (19.0%). Moreover, 8 (6.6%) patients underwent planned intended secondary TEVAR for residual extended descending aortic aneurysm. The mean duration from the TARFET to the intended secondary TEVAR was 0.9 (SD: 1.0) months. The other 15 (12.4%) patients required non-intended secondary aortic intervention with TEVAR due to type Ib endoleak in 8 (6.6%) patients, inadequate distal landing in 2 (1.7%) and progression of descending aortic aneurysm, FET stenosis and distal stent-graft-induced new entry in 1 (0.8%) patient each. Coil embolization for type II endoleak was performed in 1 (0.8%) patient. One patient required open thoraco-abdominal aortic replacement due to the progression of the descending aortic aneurysm 42 months after the TARFET.

The late mortality rate was 13.2% (16 patients). Of these, aortic-related death occurred in 4 (3.3%) patients, including non-occlusive mesenteric ischaemia in 2, stent-graft infection in 1 and aneurysm rupture in 1. Non-aortic-related death included malignancy, sepsis and pneumonia.

**Figure 3:**
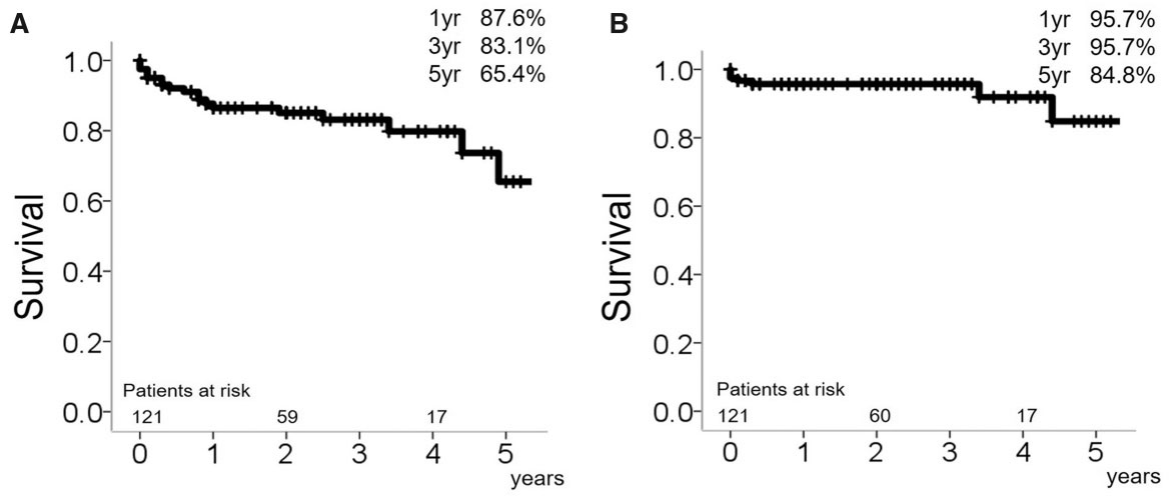
Overall long-term survival (**A**), overall aortic-related death (**B**).

**Figure 4:**
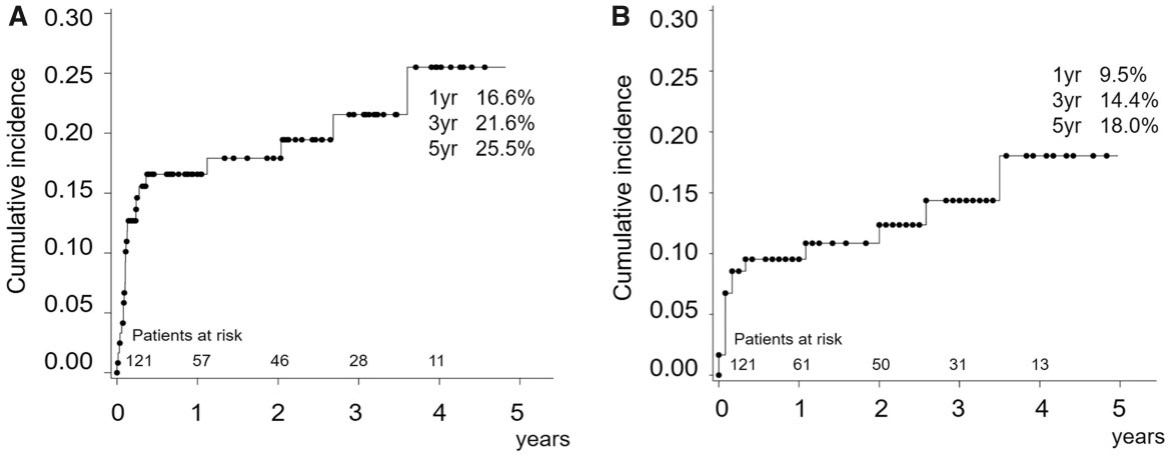
Cumulative incidence of overall secondary intervention (**A**) and cumulative incidence of non-intended secondary intervention (**B**).

The overall long-term survival rates at 1, 3 and 5 years were 87.6%, 83.1% and 65.4%, respectively, while the rates of freedom from aortic-related death at 1, 3 and 5 years were 95.7%, 95.7% and 84.8%, respectively (Fig. 3). The cumulative incidence rates of secondary intervention taking mortality as a competing risk after the TARFET at 1, 3 and 5 years were 16.6%, 21.6% and 25.5%, respectively. The cumulative incidence rates of non-intended secondary intervention at 1, 3 and 5 years were 9.5%, 14.4% and 18.0%, respectively (Fig. 4).

## DISCUSSION

The key finding of this study is that the TARFET with Frozenix for distal aortic arch aneurysms was performed with acceptable early mortality and morbidity. Moreover, the long-term outcome of the TARFET showed satisfactory survival and freedom from aortic-related death.

Surgical treatment of extensive aortic degenerative disease involving the distal aortic arch is complex and challenging for surgeons. The FET technique was first reported by Karck *et al.* in 2003 [10] and has gained popularity worldwide. The TARFET for extensive thoracic aortic diseases has received a class IIa recommendation from the Vascular Domain of the European Association for Cardio-Thoracic Surgery in 2015 [15]. The TARFET allows 1-stage repair for distal aortic arch aneurysms and is advantageous for making distal anastomosis simpler in the more proximal portion of the aortic arch with shortened circulatory arrest time [13, 17, 19]. Moreover, the TARFET provides a useful proximal landing zone to further cover the downstream diseased aorta. Although most of the previous studies have analysed the use of the TARFET for acute type A aortic dissection or mixed cohorts of acute or chronic dissection, only a few studies have focused on the use of the TARFET for degenerative aortic arch aneurysms [12, 16, 20–22]. However, aortic dissection and degenerative aortic aneurysms present substantially different pathological and morphological features. Leone *et al.* reported that the sub-analysis of their cohort for the TARFET showed a significantly older age and higher rate of concomitant preoperative diseases, including coronary artery diseases and chronic obstructive pulmonary diseases, in patients with degenerative aortic aneurysm [23]. In addition, the TARFET for thoracic aortic aneurysms presented a higher mortality rate than aortic dissection (19.3% vs 12.9%) [23]. Therefore, we focused on degenerative aortic arch aneurysms to evaluate early and long-term outcomes to demonstrate the efficacy of the TARFET and find a way to maximize the advantages of this procedure.

The 30-day mortality rate of the TARFET with Frozenix for distal aortic arch aneurysms was 2.5% in our cohort and can be considered favourable compared with the previously reported early mortality rate of 2.6–10.8% in the cohort of the TARFET for distal aortic arch diseases [1, 23, 24]. The Japan-made commercialized FET prosthesis Frozenix has 2 components: a proximal non-stent portion of the vascular prosthesis and stent portion made of oval-shaped nitinol wire secured inside the vascular prosthesis. This unique design facilitates minimizing the risk of intimal injury and providing high flexibility to conform to the curvature of the aorta [1, 20]. In addition, the delivery system of the malleable rod provides easy deployment with simple handling, and sheath markers help place the distal end of the stent at the exact target point [1, 17, 25]. Frozenix is the only available commercialized FET prosthesis in Japan at this point. Compared with single-piece devices such as Thoraflex (Vascutek, Inchinnan, Scotland, UK) or E-vita (Jotec Inc., Hechingen, Germany) used in other countries, Frozenix requires separate reconstruction of arch vessels with another branched graft. However, the separated piece of Frozenix deployment is less complicated, and the 2 flexible components of the prosthesis help conform to the complicated curvature of the aneurysmal aortic arch and to place the distal end of the FET at the exact target place of the descending aorta. We consider that the advantages of the Frozenix may contribute to better early outcomes of the TARFET for distal aortic arch aneurysms.

One of the concerns over the use of the TARFET for distal aortic arch aneurysms is spinal cord injury and paraplegia. Spinal cord injury is a devastating complication of the TARFET and affects long-term outcomes [24]. In our study, spinal cord injury occurred in 2 (1.7%) patients whose distal landing zone was below the Th8 at the beginning of our experience. Katayama *et al.* [24] reported that the distal position of the stent-graft below the Th9 and low mean blood pressure <70 mmHg are significant independent risk factors for spinal cord injury. Therefore, we changed our strategy by placing the distal end of the FET prosthesis above the Th8 level and putting every effort to achieve precise haemostasis to maintain the stable perioperative haemodynamics for the TARFET to avoid critical postoperative complications, including spinal cord injury.

While 77% of distal aortic arch aneurysms were repaired in 1-stage TARFET with Frozenix in this study, either intended or non-intended secondary aortic intervention was required in selected patients depending on the extensive aneurysm morphology. The cumulative incidence of secondary intervention after the TARFET for distal aortic arch aneurysms was 16.6% at 1 year and 25.5% in 5 years. Indeed, 1-stage repair of the distal aortic arch aneurysms is a great advantage of the TARFET; however, we tend to choose a more proximal landing of the distal end of the FET prosthesis to avoid spinal cord injury following intended secondary aortic intervention to achieve complete remodelling of the aneurysm. Of note, there was no interval death between the TARFET and intended secondary aortic intervention in this study. Intensive clinical assessment with closed imaging evaluation while waiting for secondary intervention is mandatory to avoid any interval aortic events. In addition, preoperative evaluation of aneurysm morphology with multiplanar reconstruction computed tomography and designing the appropriate diameter and length of the FET prosthesis are essential to obtain the maximum advantage of the TARFET.

Following this strategy, no spinal cord injury has been observed since then. We believe that our strategy may contribute to a lower frequency of spinal cord injuries in our institute compared with the previously reported rate that varied from 3% to 6% [1, 23, 24].

Among the 4 aortic-related late deaths, only 1 late death occurred due to the late progression of aneurysm and rupture, implying that the TARFET was effective in remodelling aortic arch aneurysm and preventing late death caused by further aneurysm rupture. The other 2 aortic-related deaths were caused by non-occlusive mesenteric ischaemia in patients with a shaggy aorta. These complications derived from the severely atherosclerotic aorta in patients with degenerative aortic aneurysms are still difficult to manage and a future issue to be solved. The overall long-term outcomes of the TARFET with Frozenix

for distal aortic arch aneurysms were acceptable, with the 3- and 5-year survival rates of 87.6% and 65.4%, respectively, which are comparable with those previously reported by Sueda *et al.* [26].

Overall, the TARFET with Frozenix is a useful treatment option for distal aortic arch aneurysms in selected patients to avoid aortic-related death. Further observation of the long-term outcome is necessary to evaluate the safety and efficacy of the TARFET with Frozenix for distal aortic arch aneurysms.

### Study limitations

This study has several limitations. First, it had a single-centre retrospective design and a small sample size. Therefore, the reproducibility of the obtained results may vary, and they may not apply to the entire population of patients with distal aortic arch aneurysms. Second, the follow-up period was relatively short to conclude the long-term durability of the TARFET with Frozenix. Nevertheless, few studies have focused on distal aortic arch aneurysm repair with the TARFET, and our experience supports the use of the TARFET with Frozenix as an important therapeutic option for distal aortic arch aneurysms. Therefore, further investigations with a larger cohort and longer follow-up periods are required.

## CONCLUSIONS

The TARFET using Frozenix for distal aortic arch aneurysms has acceptable early and long-term outcomes. Spinal cord injury and paraplegia occur less frequently, and this finding may be related the strategy of placing the FET above the Th8 level. The TARFET using Frozenix is a useful treatment option for distal aortic arch aneurysms to avoid further aortic-related death.

**Conflict of interest**: Akihiro Yoshitake is a Consultant for Japan Lifeline Co., Ltd. All other authors declare no conflicts of interest.

### Data availability statement

The data underlying this article cannot be shared publicly due to the privacy of individuals that participated in the study. The data will be shared on reasonable request to the corresponding author.

### Author contributions

**Chiho Tokunaga:** Conceptualization; Data curation; Formal analysis; Investigation; Project administration; Writing—original draft. **Yu Kumagai:** Data curation. **Fumiya Chubachi:** Data curation. **Yuto Hori:** Data curation. **Akitoshi Takazawa:** Data curation; Investigation. **Jun Hayashi:** Data curation; Investigation. **Toshihisa Asakura:** Supervision. **Ryota Ishii:** Formal analysis. **Hiroyuki Nakajima:** Supervision. **Akihiro Yoshitake:** Conceptualization; Data curation; Investigation; Supervision, Writing—review & editing.

### Reviewer information

Interactive CardioVascular and Thoracic Surgery thanks Yutaka Okita and the other anonymous reviewers for their contribution to the peer review process of this article.

## Notes

Presented at the 35th Annual Meeting of the European Association for Cardio-Thoracic Surgery, Barcelona, Spain, 13–16 October 2021.

## ABBREVIATIONS

FET: Frozen elephant trunk technique
SD: Standard deviation
TAR: Total arch replacement
TARFET: Total arch replacement using frozen elephant trunk technique
TEVAR: Thoracic endovascular aortic repair
